# Current attitudes to testicular prosthesis insertion during radical orchidectomy—An international perspective

**DOI:** 10.1002/bco2.465

**Published:** 2024-11-26

**Authors:** Anthony Emmanuel, Abi Kanthabalan, Cameron Alexander, Nikita Bhatt, Vinson Chan, Odunayo Kalejaiye, Krishna Narahari, Veeru Kasivisvanathan, Majed Shabbir

**Affiliations:** ^1^ Department of Urology Freeman Hospital Newcastle upon Tyne UK; ^2^ Department of Urology Worcestershire Royal Hospital Worcester UK; ^3^ Department of Urology Royal Bolton Hospital Greater Manchester UK; ^4^ Department of Urology Norfolk and Norwich University Hospital Norwich UK; ^5^ School of Medicine, Faculty of Medicine and Health University of Leeds Leeds UK; ^6^ Department of Urology North Bristol NHS Trust Bristol UK; ^7^ Department of Urology, University Hospital of Wales, Cardiff and Division of Cancer & Genetics Cardiff University UK; ^8^ Division of Surgery and Interventional Science University College London London UK; ^9^ Department of Urology Guy's & St. Thomas' Hospital, and Faculty of Life Sciences & Medicine, King's College London London UK

**Keywords:** implantation, radical orchidectomy, survey, testicular cancer, testicular prosthesis

## Abstract

**Objectives:**

This study aimed to assess current international clinician practices, attitudes and barriers related to testicular prosthesis implantation in patients with testicular cancer at the time of radical inguinal orchidectomy.

**Methods:**

An international online survey of urologists who perform radical orchidectomy for testicular cancer was developed. The recruitment process used social media and the emailing lists of national urological societies. Responses were collected between 10 February 2021 and 31 May 2021. The primary outcome was the proportion of urologists who always offered testicular prosthesis implantation to patients undergoing radical orchidectomy. Secondary outcomes included the reasons for not offering testicular prosthesis implantation.

**Results:**

A total of 393 respondents took part in the online survey; of these, the majority were from the UK (66%), with the remaining international respondents (34%) from six different continents. Urologists (53%) reported they always offer testicular prosthesis implantation. Of those that offered testicular prosthesis implantation, 28% did so as a secondary procedure after radical orchidectomy, rather than the time of radical orchidectomy (72%). The most frequently selected reasons for not offering testicular prosthesis implantation included concerns about delaying chemotherapy (41%), infection (33%), impaired cosmesis (17%) and lack of availability (17%).

**Conclusion:**

Despite evidence confirming the safety and the psychological benefit of testicular prosthesis implantation during radical orchidectomy, current international practice suggests just over half of urologists always offer this to their patients. Increased clinician awareness of the low risk of complications and high patient satisfaction may act to reduce the perceived barriers in offering testicular prosthesis implantation.

**Abbreviations**
RO: radical orchidectomy
TC: testicular cancer
TP: testicular prosthesis
TPI: testicular prosthesis implantation

## 1 INTRODUCTION

The excellent long‐term cancer‐specific survival rates seen with modern management strategies for localised testicular cancer (TC) has led to an increased focus on long‐term survivorship and quality of life issues for men that undergo radical orchidectomy (RO).^1^, ^2^ Testicular prosthesis (TP) has been in use for over 80 years and has been shown to relieve the psychological burden of testicular loss and to improve male body image perception, with high rates of patient satisfaction reported. These issues are of increasing importance in an era of increasing social media use and male body dysmorphia.^3^, ^4^, ^5^

It has been established in large, contemporary case series that testicular prosthesis implantation (TPI) at the time of RO does not increase peri‐operative complications, length of stay, re‐admission and need for further surgery.^6^ Furthermore, TP‐related complications have been shown not to cause a delay in chemotherapy or radiotherapy, which is a commonly cited reason by clinicians for not offering a TPI during RO.^7^ These findings are reflected in international guideline recommendations but, in spite of this, there appears to have been limited improvements in offering TPI at the time of RO.^2^, ^8^ A recent patient questionnaire from Nichols et al.^9^ in 2019 found that 42% of TC patients that underwent RO without TPI reported that this had never been offered to them.^6^, ^8^ A relative lack of contemporary evidence means that there is a poor understanding of international clinician decision making surrounding TP use with RO, and in particular of potential perceived barriers to offering TPI. The objective of this study was to assess current practice, attitudes and barriers related to TPI at the time of RO in order to improve compliance with guidelines in this practice area.

## 2 SUBJECTS AND METHODS

The British Association of Urological Surgeons (BAUS) and British Urology Researchers in Surgical Training (BURST) devised a joint international web‐based survey, which was divided into two components surrounding care at the time of RO. These two components were TPI and fertility assessment/preservation, of which the results of the latter have been recently published.^10^ This article primarily focuses on TPI, and the results were reported using the Checklist for Reporting Results of Internet E‐Surveys (CHERRIES).^10^, ^11^

### 2.1 Study design

The survey was obtained using a probability list–based sampling approach using email addresses to target urologists via national urological societies in the United Kingdom and a non‐probability sampling technique to reach international urologists using an unrestricted self‐selected survey method by including a link to the survey on the social media platform, X (formerly known as ‘Twitter’).^12^

The survey was anonymised (unless respondents volunteered their names for acknowledgment purposes), and responses were collected and managed using REDCap (Research Electronic Data Capture) hosted by the University College London. Data access was allocated in advance of study commencement to only three authors (AE, AK, NB), and the scope of questions and format were drafted by AE, AK, and MS and then revised by all authors. The web‐based survey was exempt from requiring ethical approval, although informed consent was obtained from the respondents within the survey. Responses from the web‐based survey were collected over a 7‐week period from 10 February 2021 to 31 May 2021.

### 2.2 Inclusion and exclusion criteria

Respondents were eligible to complete the survey voluntarily, if they were a practicing urologist or urologist in training that routinely performed RO for TC. No restrictions were made by country of practice, and there were no financial incentives offered for completion of the survey. The REDCap platform ensured that only a single entry could be recorded for each allocated username. Data was also collected on participant clinician grade place of work, as well as the timing of survey completion which facilitated the identification of potential duplicate records. All responses were screened for inclusion eligibility by two authors (AE and AK) from the study group.

### 2.3 Survey content

The format of questions primarily required the respondent to select the single best or most applicable answer from a short list. The survey consisted of 50 questions and included data on participant details. To assess clinician attitudes and perceived barriers to TPI, the remaining questions incorporated the use of a Likert scale.

### 2.4 Outcomes

#### 2.4.1 Primary outcome

1. The proportion of urologists who always offer TP to patients with TC undergoing RO

#### 2.4.2 Secondary outcomes

1. The proportion of urologists who never offer TPI to patients with TC undergoing RO
2. The categorised reasons for urologists not offering TP to TC patients undergoing RO
3. The proportion of urologists who offer upfront TPI at the time of RO
4. The proportion of urologists who offer delayed TPI within 12 months of RO
5. The proportion of patients who accept a TP when offered

### 2.5 Analysis

Following data cleaning, the use of descriptive analysis was undertaken to determine the key cohort parameters and study outcomes. Where respondents submitted a survey which contained missing data, analysis was still performed if valid responses had been recorded for individual parameters. Where data was missing for individual parameters, then the denominator was adjusted accordingly.

## 3 RESULTS

### 3.1 Respondent characteristics and response rate

**FIGURE 1:**
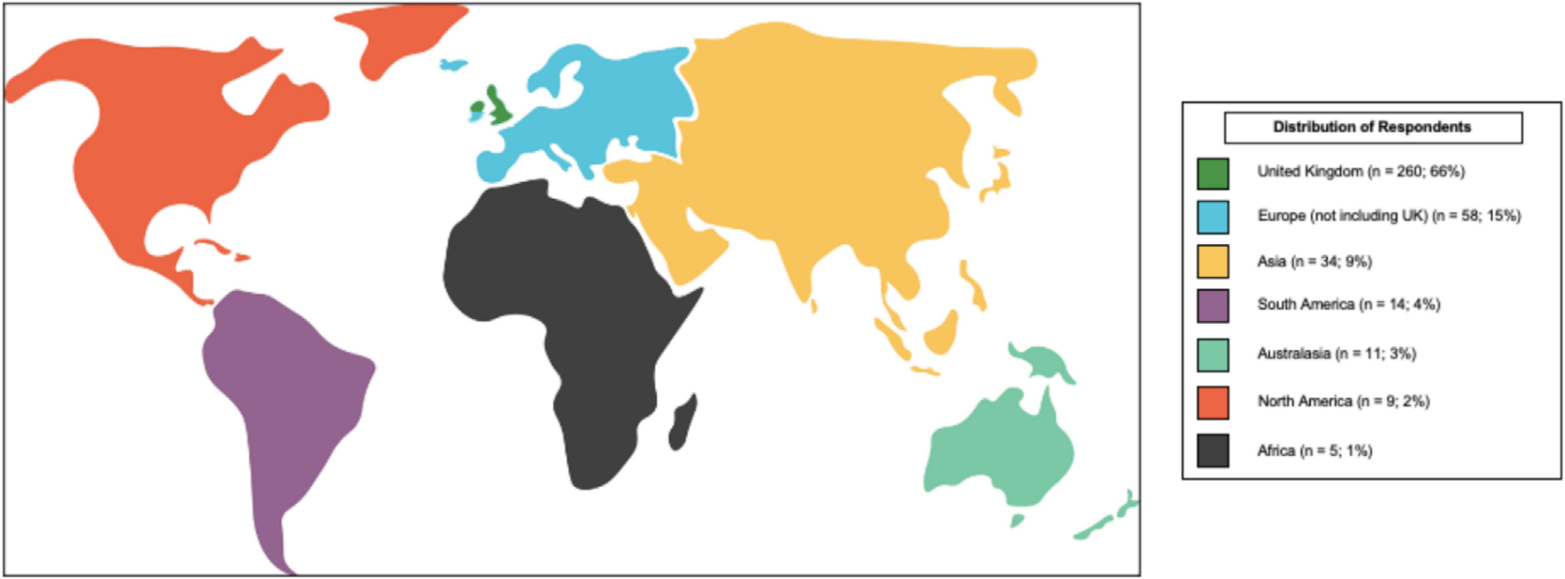
Distribution of survey respondents.

A total of 393 respondents took part in the survey: 260/393 (66%) were from the UK and 133/393 (34%) were international from six different continents which included 45 countries. The largest proportions of non‐UK respondents were from Europe (58/393; 15%) and Asia (34/393; 9%) (Figure 1).

Of the 393 respondents who took part in the survey, 354 (90%) completed all questions. The majority of respondents were fully qualified urologists (consultants/attendings/associate specialists/professors) (68%, 269/393), based in a tertiary centre (248/391; 63%) and had no subspecialty interest in andrology (283/392; 72%). Overall, clinicians most frequently performed between 5 and 10 ROs within a typical 12‐month period (182/392; 46%) closely followed by those who performed less than five ROs within the same time period (175/392; 45%) (Table 1).

**TABLE 1:** Respondent characteristics.

	All respondents (%)	UK respondents (%)	International respondents (%)
Total number of respondents	393 (100)	260 (66)	133 (34)
Grade of clinician			
Fully qualified urologist			
Professor of urology/consultant/attending	246 (62)	173 (67)	73 (55)
Associate specialist	23 (6)	4 (1)	19 (14)
Urologist in training			
Urological trainee/resident	103 (26)	68 (26)	35 (26)
Urology fellow	21 (5)	15 (6)	6 (5)
Affiliated institution			
Tertiary care (teaching hospital or regional referral centre)	248 (63)	143 (55)	105 (79)
Secondary care (district general hospital or community hospital)	143 (36)	115 (44)	28 (21)
No response	2 (1)	2 (1)	0
Subspecialty interest in andrology			
No	283 (72)	199 (77)	84 (63)
Yes	109 (28)	60 (23)	49 (37)
No response	1 (<1)	1 (<1)	0
Individual annual RO volume (mean cases/year)			
<5	175 (45)	124 (48)	53 (40)
5–10	182 (46)	124 (48)	58 (44)
>10	33 (8)	12 (4)	21 (16)
No response	1 (<1)	0	1 (<1)

When compared to UK respondents, a higher proportion of international respondents were working in tertiary care centres (79% vs 55%), had a subspecialty interest in andrology (37% vs 23%) and performed >10 ROs per year (16% vs 4%). (Appendix 1).

### 3.2 Outcomes

**FIGURE 2:**
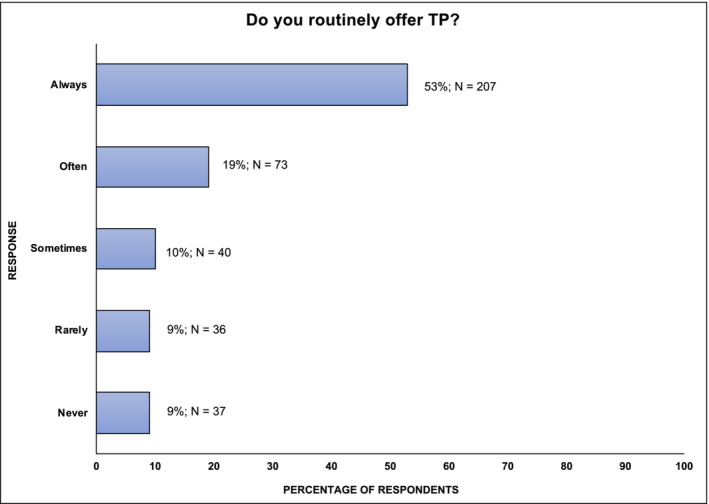
International practices for offering TP to patients with TC undergoing RO. TC, testicular cancer; TP, testicular prosthesis; RO, radical orchidectomy.

Fifty‐three percent (207/393) of respondents reported that they ‘always’ offered a TP on a routine basis to patients undergoing RO for TC. In contrast, 9% (37/393) reported they would ‘never’ offer this, with similar proportions reporting that they would do this only ‘rarely’ (9%; 36/393) or ‘sometimes’ (10%; 40/393) (Figure 2).

When compared to urologists in training, a higher proportion of fully qualified urologists would always offer TPI (56% vs 45%). The status of sub‐specialty interest in andrology did not appear to make a notable difference in the likelihood of always offering TPI; 55% of those with a sub‐specialty interest in andrology always did compared to 52% of non‐andrologists. There was geographical variability regarding the likelihood of respondents never offering TPI; this was lowest in South America (0%), UK (5%), Europe (9%) and Australasia (9%), with higher levels seen in North America (18%), Asia (41%) and Africa (60%) (Appendix 1).

**FIGURE 3:**
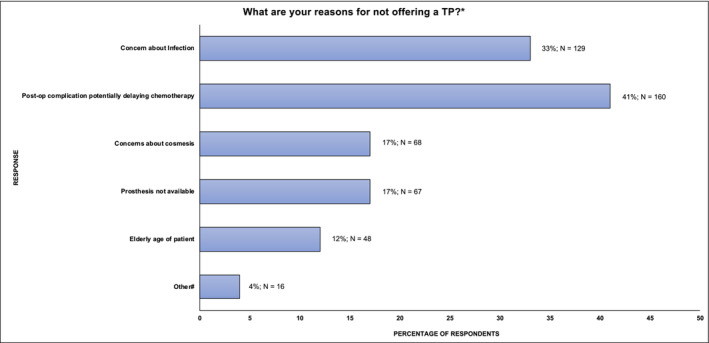
International respondent reasons for not offering TP (*All respondents were permitted to select all reasons that apply. #Other reasons given include not required by patient or main concerns [*n* = 7], pain [*n* = 2], prohibitive cost [*n* = 3], previous surgical procedure such as a mesh repair [*n* = 2], patient not psychologically ready for TP [*n* = 2]). TP, testicular prosthesis.

The two most frequently selected reasons for not offering TPI were concerns regarding delaying chemotherapy due to post‐operative complications (160/393; 41%) and post‐operative infection (129/393; 33%) (Figure 3). A notable proportion of respondents reported that TPI was not offered due to lack of availability (17%; 67/393) and perceived poor cosmesis (17%; 68/393). Where lack of availability was selected as the reason for not offering TPI, this was applicable to a larger proportion of respondents from Africa (80%; 4/5), South America (64%; 9/14), Asia (62%; 21/34) and North America (56%; 5/9) (Appendix 1).

**FIGURE 4:**
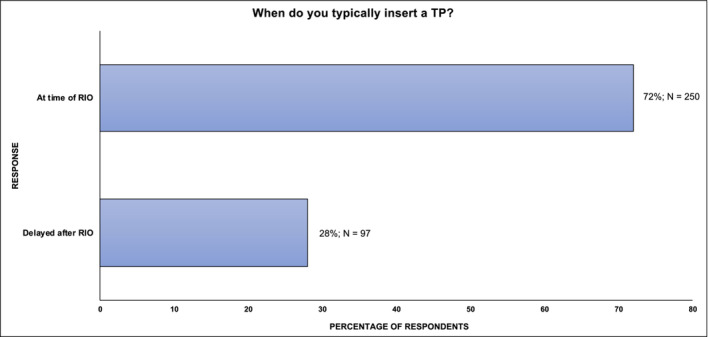
Respondent preferred timing of performing a TPI if offered. TPI, testicular prosthesis implantation.

The majority of urologists who offered TPI performed this upfront at the time of RO (72%; 250/347 rather than as a delayed, separate procedure within 12 months (28%; 97/347) (Figure 4). Comparisons of international practice demonstrated broadly similar approaches to this, but the highest proportion of upfront TPI was seen in North America (88%; 7/8) and the UK (75%; 182/244) (Appendix 1).

**FIGURE 5:**
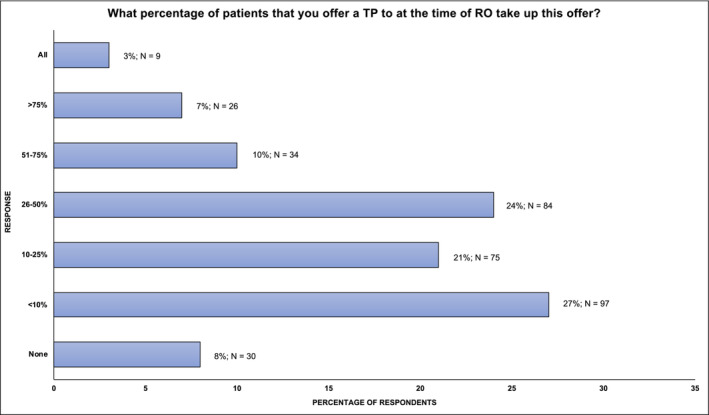
Patient acceptance of TP when offered at time of RO. TP, testicular prosthesis; RO, radical orchidectomy.

When asked about the likelihood of patient acceptance of a TP when offered, most urologists (81%; 286/355) felt this was accepted by half or less (≤50%) of patients. Three percent (9/355) of urologists reported that TPI would be accepted by all patients in their practice in comparison to 8% (30/355) who felt this offer was never accepted (Figure 5).

## 4 DISCUSSION

Our survey having amalgamated data from 45 countries has provided a unique insight into contemporary international practices, attitudes and variations regarding the use of TPI and fertility assessment/preservation in TC patients undergoing RO. As the latter is discussed in another paper by our group, this manuscript focuses on the utilisation of TPI internationally, and to the best of our knowledge, no previous surveys have assessed clinician (rather than patient) attitudes and perceived barriers towards TPI.

The potential psychological benefits of TPI to TC patients have been well described, and both the European Association of Urology and American Urological Association (AUA) recommend that TP should be offered to all patients prior to RO.^2^, ^7^, ^8^ In spite of this, only 53% of respondents in this study always offered TPI to all patients undergoing RO. Whilst this was slightly higher in some developed countries (UK; 61%), the last decade does not appear to have demonstrated the sustained rise in patients being offered TPI that has been reported in previous studies.^6^, ^13^ In two previous UK‐based studies, Robinson et al. reported that 60.8% of men were offered TPI in 2009^6^ and Adshead et al. reported that two‐thirds of men were offered TPI in 2001.^13^

This study has demonstrated that the perceived risk of delaying future chemotherapy (due to postoperative complications) or infection remain the most common reasons for clinicians not offering TPI. These concerns are also likely to account for the significant proportion of urologists in this cohort (28%) who choose to offer TPI in a delayed fashion as a separate operation. Whilst these practices aim to reduce patient‐related complications, they are not supported by existing evidence. Robinson et al. have previously demonstrated that concurrent TPI does not increase complication rate of RO in terms of length of stay, re‐admission or the need for the further surgery in a cohort of more than 900 patients,^6^ and Musi et al. demonstrated that, even where complications occurred, this did not lead to delay in chemotherapy or radiotherapy.^7^ Musi et al. reported that the risk of TP‐related infection requiring surgical removal in patients undergoing adjuvant chemotherapy was extremely low at 0.72%.^7^ It is also possible that clinician biases related to perceived TP‐related complications influence pre‐operative counselling and patient choice and in turn explain the relatively low proportion of patients that accept TPI in this cohort (<50%) and in other studies.^6^, ^14^

Although 17% of clinicians in this cohort cited poor cosmesis as a barrier to offering TPI, this does not correlate with patient reported outcomes from other studies, which demonstrate high levels of patient satisfaction with TPI; Clifford et al.^15^ and Ramos et al.^16^ reported patient satisfaction was good or excellent in 98% and 83% of cases, respectively. The estimated cost of TP at $2500–$3000,^17^ however, is likely to explain some of the observed geographical variations in practice, with a lower proportion of clinicians always offering TPI in developing countries or those with insurance‐based health care systems. Nichol et al. for example has demonstrated that a higher proportion of men in North America (58%) were offered TPI than in this study.

It is important to recognise the presence of methodological limitations within our study. The use of global social media platforms, such as ‘X’, formerly known as ‘Twitter’ and national urological association emailing lists to advertise and distribute this survey means that only urologists that were reachable via these methods could participate. This represents a likely source of selection bias, and the distribution of the survey in English will have likely reduced participation from urologists in non‐English speaking countries. Further to this, it is not possible to comment on the precise number of potentially eligible participants that would have been reached by these methods or to offer any detailed geographical or demographic data on those urologists who received the survey but chose not to participate.

Whilst this survey includes broad international involvement, the bias towards UK responses (66%) and the relative sparsity of data from other single countries presents significant challenges in making meaningful comparisons in clinical practice. In particular, there appears to be under‐representation of North America (*n* = 9) and Africa (*n* = 5). (Appendix 1).

This survey is also limited in its ability to explore broader aspects of patient opinion and perspectives on TPI, as only urologists were eligible to participate. The scope of this survey was primarily to understand the barriers that exist at the level of the decision making of the individual clinician, and therefore, there may be other patient‐specific barriers that will have not been well explored in this survey.

An important consideration related to the specific phrasing used in the survey is that urologists were asked to comment in what proportion of cases they would ‘always’ offer prosthesis to a patient undergoing RO for TC. This question was designed to capture what would be considered to be routine practice in a typical TC patient, but it may not take account of other rare clinical nuances, when TPI may be considered less appropriate. This may include the elderly patient population or where active infection is considered. It may therefore be the case that the primary outcome of 53% of urologists ‘always’ offering TP creates an exaggerated sense of poor TP use and counselling and that 72% (which includes 19% of those who report offering this ‘often’) is more reflective of typical every day practice.

Clinical implications of this work are that now that current practice patterns have been demonstrated, this allows for improvement in compliance with current guidelines with respect to TPI at time of RO. The study emphasises to clinicians the rationale for offering TP, current guidelines and associated clinical outcomes. Research implications include highlighting potential targets for implementation work in clinician behaviour modification.

## 5 CONCLUSION

International urological guidelines recommend that TP should be offered to all men undergoing RO, but this study has demonstrated that just over half of clinicians currently always offer this. TPI at the time of RO reduces the psychological burden from testicular loss and improves male body image perception. Increased clinician education and awareness of the low risk of complications and high patient satisfaction may address some of the perceived barriers in offering TPI.

## AUTHOR CONTRIBUTIONS

**A Emmanuel:** project and development, data collection, data analysis, manuscript writing, review and editing of manuscript. **A Kanthabalan:** project and development, data collection, data analysis, manuscript writing, review and editing of manuscript. **C Alexander:** project and development, reviewing and editing of manuscript. **N Bhatt:** project and development, reviewing and editing of manuscript. **V Chan:** project and development, reviewing and editing of manuscript. **O Kalejaiye:** project and development, reviewing and editing of manuscript. **K Narahari:** project and development, reviewing and editing of manuscript. **V Kasivisvanathan:** project and development, reviewing and editing of manuscript. **M Shabbir:** project and development, reviewing and editing of manuscript.

## CONFLICT OF INTEREST STATEMENT

The authors declare no conflicts of interest.

## Appendix

Subgroup analysis of survey responses

**Table:**

	UK	Europe	Asia	S. America	Australasia	N. America	Africa
Total number of respondents	260	58	34	14	11	9	5
Individual annual RIO volume (mean cases/year)							
<5	124 (48%)	23 (39%)	14 (41%)	2 (14%)	4 (36%)	5 (55%)	5 (100%)
5–10	124 (48%)	25 (43%)	17 (50%)	6 (43%)	6 (55%)	3 (33%)	‐
>10	12 (4%)	10 (18%)	2 (6%)	6 (43%)	1 (9%)	1 (11%)	‐
No response	‐	‐	1 (3%)	‐	‐	‐	‐
Do you routinely offer a testicular prosthesis?							
Never	12 (5%)	5 (9%)	14 (41%)	‐	1 (9%)	2 (18%)	3 (60%)
Rarely	16 (6%)	7 (12%)	5 (15%)	2 (14%)	1 (9%)	2 (18%)	2 (40%)
Sometimes	22 (8%)	8 (14%)	5 (15%)	1 (7%)	2 (18%)	1 (11%)	‐
Often	51 (20%)	12 (21%)	4 (12%)	2 (14%)	3 (27%)	1 (11%)	‐
Always	159 (61%)	26 (44%)	6 (17%)	9 (64%)	4 (36%)	3 (33%)	‐
When do you typically insert a testicular prosthesis?							
At time of RIO	182 (75%)	36 (68%)	7 (50%)	9 (69%)	8 (73%)	7 (88%)	1 (50%)
Delayed (after RIO)	62 (25%)	17 (32%)	7 (50%)	4 (31%)	3 (27%)	1 (12%)	1 (50%)
What percentage of patients that you offer a testicular prosthesis at the time of RIO take up this offer?							
None	37 (14%)	4 (7%)	4 (12%)	‐	1 (9%)	‐	1 (20%)
<10%	68 (26%)	7 (12%)	8 (24%)	6 (43%)	4 (36%)	4 (44%)	1 (20%)
10%–25%	61 (23%)	8 (14%)	2 (6%)	2 (14%)	1 (9%)	1 (11%)	‐
26%–50%	55 (21%)	10 (17%)	2 (6%)	4 (29%)	4 (36%)	2 (18%)	‐
51%–75%	23 (9%)	5 (9%)	2 (6%)	1 (7%)	‐	‐	‐
>75%	15 (6%)	12 (21%)	2 (6%)	‐	‐	‐	‐
All	1 (1%)	6 (10%)	‐	1 (7%)	‐	‐	‐
No response	‐	6 (10%)	14 (41%)	‐	1 (9%)	2 (18%)	3 (60%)
Reasons for not offering a testicular prosthesis							
Concerns about Infection	96 (37%)	19 (33%)	8 (24%)	2 (14%)	1 (9%)	2 (18%)	‐
Concerns about post‐operative complication delaying chemotherapy	131 (51%)	15 (26%)	5 (15%)	3 (21%)	4 (36%)	1 (11%)	1 (20%)
Concerns about cosmesis	39 (19%)	8 (14%)	2 (6%)	1 (7%)	3 (27%)	4 (44%)	‐
Prosthesis not available	9 (3%)	15 (26%)	21 (62%)	9 (64%)	3 (27%)	5 (56%)	4 (80%)
Elderly age of patient	28 (11%)	15 (26%)	1 (3%)	2 (14%)	1 (9%)	1 (11%)	‐
Other	60 (23%)	7 (12%)	5 (15%)	‐	1 (9%)	‐	‐
